# The Growth of Bone

**Published:** 1912-06

**Authors:** 


**The Growth of Bone.** By WILLIAM MACEWEN, F.R.S. Pp. 210. Glasgow: James Maclehose and Sons. 1912.

This little book is one of extraordinary interest. In fact, it may be described as almost sensational in its iconoclastic novelty. Its distinguished author proves by a series of simple experiments that the commonly accepted teaching about the growth and vitality of bone depending upon the periosteum is quite fallacious.

It is shown how when a sub-periosteal resection of bone is made, no new bone is formed from the intact periosteum; that periosteal flaps, however they are formed, will not produce bone; that new bone is rapidly formed from the cut ends of the shafts of long bones from which a piece has been removed; that total removal of the periosteum from bone does not impair its vitality; and that pieces of bone cut up into small fragments and transplanted can and do grow into firm, bony masses.

The periosteum is nothing more than a limiting membrane, and the living osteoblasts in the bone itself are the dominating vital factors in bone growth.

The regeneration of the shaft of a necrosed bone as a sub-periosteal tube is regarded merely as being the result of the increased activity of osteoblasts driven out from the inflamed bone into the loose connective tissue between the periosteum and the shaft.

All the experiments are beautifully illustrated, and the most sceptical reader cannot fail to be convinced by the simple demonstrations they afford. It would be difficult to exaggerate the practical importance of this work.

